# Renal Risk Medication Quick Guide to Aid Pharmacist-Led Medication Review in Frail Hospitalized Geriatric Patients: A Multicenter Exploratory Study

**DOI:** 10.3390/healthcare14091245

**Published:** 2026-05-05

**Authors:** Joo Hanne Poulsen Revell, Anne Byriel Walls, Trine Rune Høgh Andersen, Faruk Coric, Ulla Hedegaard, Charlotte Olesen, Anita Buch Grann Press, Lene Vestergaard Ravn-Nielsen, Lisa Greve Routhe, Lene Juel Kjeldsen

**Affiliations:** 1 The Hospital Pharmacy Research Unit, University Hospital Sønderjylland, 6200 Aabenraa, Denmark; 2 The Hospital Pharmacy, University Hospital Sønderjylland, 6200 Aabenraa, Denmark; 3 The Department of Regional Health Research, University of Southern Denmark, 5000 Odense, Denmark; 4 The Capital Region Pharmacy, 2730 Herlev, Denmark; 5 Department of Drug Design and Pharmacology, Faculty of Health and Medical Sciences, University of Copenhagen, 2900 Copenhagen, Denmark; 6 Region Zealand Hospital Pharmacy, 4700 Roskilde, Denmark; 7 Hospital Pharmacy Funen, Odense University Hospital, 5700 Odense, Denmark; 8 Clinical Pharmacology, Pharmacy and Environmental Medicine, Department of Public Health, University of Southern Denmark, 5000 Odense, Denmark; 9 Hospital Pharmacy Central Denmark Region, 8000 Aarhus, Denmark; 10 The Hospital Pharmacy in the North Denmark Region, 9260 Aalborg, Denmark; 11 Department of Clinical Medicine, Aalborg University, 9260 Aalborg, Denmark

**Keywords:** chronic kidney disease, drug-related problems, exploratory study, impaired renal function, pharmacist-led medication reviews, renal risk medications, risk assessment quick guide

## Abstract

**Background/objectives:** Chronic kidney disease (CKD) affects approximately 800 million individuals worldwide and poses a growing health challenge. To support safer prescribing practices, our research group developed a renal risk medication quick guide (RRMQG), which provides recommendations for the 50 most prevalent renal risk medications (RRMs). This study explored the usefulness of implementing the RRMQG in Danish hospital settings. **Methods:** In this multicenter pilot study conducted across six Danish hospitals, 28 clinical pharmacists applied the RRMQG during medication reviews of frail, geriatric patients with renal impairment from May to October 2023. Useability was explored through structured surveys completed by the pharmacists. **Results:** Among 182 patients, 378 RRMs were detected, and 14% of these were associated with potential drug-related problems (DRPs). The RRMs were distributed across 35 of the 50 from the RRMQG, and patients received an average of two RRMs each. Most pharmacists found the RRMQG manageable for implementation in practice. **Conclusions:** The RRMQG was useful and manageable for implementation in practice in Danish hospitals, particularly due to its medication-specific recommendations. However, adjustments to the RRMQG may be beneficial by, for example, adding opioids or context-relevant medications to meet individual needs.

## 1. Introduction

More than one-third of all medications, or their active metabolites, are renally excreted and can be classified as renal risk medications (RRMs) [1]. Impaired kidney function results in prolonged excretion of RRMs, which increases the risk of adverse drug reactions, acute kidney injury (AKI), hospital admissions and mortality [2]. Accordingly, these medications require adjustment of dose or administration frequency, when renal function is compromised. However, current methods to detect inappropriate prescribing of RRMs are unsystematic and insufficient for RRMs.

Chronic kidney disease (CKD) affects approximately 800 million individuals worldwide and represents a significant and growing public health concern [3]. CKD is defined by structural and functional abnormality of the kidneys, which persists for at least three months [4]. However, an estimated glomerular filtration rate (eGFR) below 60 mL/min per 1.73 m^2^ has been proposed to be the key criterion for diagnosis of CKD [4,5,6]. Further, kidney function naturally declines with age [7], and additional factors like diabetes, cardiovascular diseases, and inflammation contribute to an accelerated GFR decline [8], which is progressive in CKD [8]. In addition, multimorbidity and polypharmacy are prevailing with age, with nearly 40% of the elderly worldwide using five or more medications [9]. The threshold for defining elderly patients as geriatric is commonly set at 65 years of age and older. However, significant health issues, such as kidney impairment, may not manifest until later in life. Consequently, frailty has become a key component in geriatric assessment. Frailty is characterized as a state of increased vulnerability resulting from diminished physiological reserves, and it is frequently observed in older adults. As a central concept in geriatric medicine, frailty is used to identify individuals at higher risk for adverse health outcomes, including adverse drug events, hospitalization, and mortality [10]. These events may be a consequence of diminished physiological reserves leading to alterations in the pharmacokinetic and pharmacodynamic properties of medications, which makes elderly patients more susceptible to dosing errors [11].

Our research group developed a renal risk medication quick guide (RRMQG) providing recommendations for the 50 most prevalent medications across healthcare sectors in Denmark [12]. The RRMQG was developed to explicitly focus on assessing renal risk medications and it was designed to provide a readily available tool for clinical pharmacists when evaluating RRMs during medication reviews in hospitals [12]. This guide was created based on data from the Renal Drug Database (which contains monographs written by clinical renal pharmacists, providing dose adjustment recommendations based on disease characteristics and validated under the governance of the UK Renal Pharmacy Group) and the Danish drug monography Pro-medicin.dk (a national drug monography primarily based on Summaries of Product Characteristics (SPCs) and frequently used in daily practice to support medication-related decision making) [12].

Despite the potential benefits of such a RRMQG to assist clinical pharmacists in conducting medication reviews in Danish hospitals, the prevalence of suboptimal prescription of RRMs has not yet been studied in Denmark. Thus, this study aimed to explore the useability of the RRMQG when applied to elderly patients with frailty and impaired renal function treated with RRMs at Danish hospitals.

## 2. Materials and Methods

This study was conducted by the Danish Hospital Pharmacy Research Network (DanHoPR Network), and hospital pharmacies were invited to participate. An invitation was sent to the hospital pharmacy managers, and hospital pharmacists who were interested participated in an introduction session to this study and a three-hour teaching session in impaired renal function and RRMs. Twenty-eight clinical pharmacists from six out of eight hospital pharmacies across Denmark’s five regions participated in data collection.

### 2.1. Setting

In Denmark, the healthcare system is organized into five regions, each being responsible for hospital care, including emergency services, and eight hospital pharmacies provide medicine to the hospitals. The Danish healthcare system is based on the principles of universal and equal access to healthcare for all citizens and is financed by general taxes to provide access free of charge for the residents. During hospital admission, several healthcare professionals, including medical doctors, nurses, and clinical pharmacists, collaborate to provide specified treatment and care. The primary patient-related task for clinical pharmacists is to conduct medication reviews [13]. Pharmacist-led medication reviews may result in interventions, such as leading to changes in dosing regimen, choice of drug or discontinuation of treatment [14,15,16]. At the time of this study, no national clinical decision support system concerning RRMs and impaired renal function was well implemented in Danish hospitals.

### 2.2. Design and Patients

The design of this study is framed as a service evaluation study where the useability and implementation of the RRMQG are explored.

Data collection was performed during the period from May to October 2023. A structured data collection form was used to document information on age, sex, body mass index, eGFR, frailty, comorbidities, and RRMs including substance, dose, and frequency as well as potential intervention. Estimated GFR was documented from the electronic system on admission, at medication review, and at discharge from the hospital. In addition, information about hospital readmission or mortality within 30 days of discharge was collected from the electronic patient journal. Twenty-eight clinical pharmacists from six out of eight hospital pharmacies in Denmark collected data in relation to performing medication reviews at their referring units (medical, surgical and emergency departments). Prior to data collection, clinical pharmacists were trained on how to collect data from an online course.

Data were collected using Research Electronic Data Capture 15.5.36 (REDCap). Inclusion criteria were the following:

1. Age ≥ 65 years.
2. eGFR below 60 mL/min/1.73 m^2^ during a period of at least 90 days prior to inclusion.
3. Treatment with at least one RRM mentioned from the RRMQG.
4. Presenting with at least one criterion from the Clinical Frailty scale [17]: unintentional weight loss (4–5 kg or 5% over the last year); increasing fatigue; fatigue during daily activities; deterioration in activities of daily living; easy fatigue and decreased energy or decreased appetite; low muscle strength, weak grip strength, or slow walking; vulnerability (unable to take care of oneself); cognitive decline.
5. Multimorbidity with 2 or more chronic diagnoses.

Patients were excluded at inclusion if no plasma creatinine measurement was available within the 90 days preceding hospital admission and if AKI was detected upon inclusion in accordance with KDIGO criteria. AKI was defined as an increase or decrease in eGFR by more than 50% detected in the patient upon admittance, during hospitalization or at discharge.

### 2.3. Power Calculation and Statistical Analyses

Due to this study being an exploratory pilot study, no power calculation was performed, and consequently no formal sample size estimation was conducted for the number of patients included. However, χ^2^-test was performed to qualify differences found among categorical variables.

### 2.4. Outcome Measure—Application of RRMQG

RRMs prescribed to included patients were assessed for adherence to the recommendations in the RRMQG [12]. Non-adherence was considered as a potential DRP.

The RRMQG was developed by the research group and comprised the 50 most prevalent medications across healthcare sectors [12]. The RRMQG also contained recommendations for adjustment to prescriptions of each medication on the list for patients with renal impairment [12].

### 2.5. Categorization of DRPs Associated with RRMs

Three assessment pharmacists identified all RRMs and performed a post-categorization model of potential DRPs using the Pharmaceutical Care Network Europe (PCNE) (model V9.1) [18]. Any disagreements were discussed until consensus was reached. From there, RRMs were divided into three categories: PCNE-DRP (non-adherence to RRMQG, meaning an identified discrepancy between the quick guide recommendation and the prescribed dosing regimen, which had the potential to lead to a DRP); monitoring (an identified RRM adhered to the recommendation in RRMQG (no PCNE-DRP classification was present), but monitoring activity was recommended based on RRMQG); and “no recommendation” (the clinical pharmacists reported adherence to the RRMQG and no recommendation was provided. However, the assessment group was unable to confirm adherence to RRMQG due to missing information, such as dose, dosing interval, and indication). Consensus among the assessment pharmacists was achieved for all categorizations.

### 2.6. Clinical Pharmacist Evaluation

Useability and impact of using the RRMQG by clinical pharmacists was assessed by a post-study survey. The pharmacists were asked to evaluate the RRMQG in terms of conducting pharmacist-led medication regarding usability, quality improvement and implementation in practice, alongside their levels of experience with clinical services in hospital pharmacies (see Supplementary File S1).

### 2.7. Ethics Approval

This study was a non-interventional Danish study, and no ethical approval was needed under Danish legislation. Each hospital pharmacy obtained the required approval to conduct this study in accordance with regional policy. The Hospital Pharmacy, University Hospital Sønderjylland, obtained approval from The Region of Southern Denmark’s repository to store all data in accordance with regional policy (record no. 23/19380).

## 3. Results

### 3.1. Data Collection

A total of 182 patients were included in this study. Patients were admitted to medical departments (43%, *n* = 78), surgical departments (26%, *n* = 48), and emergency departments (31%, *n* = 56). Following hospital discharge, 28% (*n* = 52) were readmitted to the hospital, and 18% (*n* = 33) died within 30 days.

### 3.2. Demographic Data

Patients had a median age of 82.0 years, with 52% being female (Table 1). Body mass index (BMI) was calculated for 163 of the 182 included patients, with a median BMI of 25.1 kg/m^2^. At the time of medication review, the median estimated glomerular filtration rate (eGFR) was 34 mL/min/1.73 m^2^, and the median number of comorbidities was two, with cardiovascular diseases being the most prevalent, affecting 92% of patients (Table 1).

### 3.3. Renal Risk Medications

Data on RRMs listed in the RRMQG were collected for all 182 patients, resulting in 469 RRMs. However, 91 RRMs were excluded due to the medications not being sufficiently specified. Thus, for 157 patients with a complete or partial specification of medications from the RRMQG, a total of 378 RRMs were identified (Table 2). Consistent with the high prevalence of cardiovascular diseases in this patient group, medications categorized as ATC C (cardiovascular system) were most common (48.9%). Notably, diuretics (ATC C03) were particularly abundant among these patients, exhibiting an eGFR below 60. Additionally, anticoagulants (ATC code B), used for stroke prevention in various cardiovascular conditions, including atrial fibrillation and post-cardiac stroke, were also prevalent.

### 3.4. Adherence to Recommendations of the RRMQG

A total of 378 specified RRMs (including 14 paused medications) were assessed for adherence to the recommendations of the RRMQG (Table 3). Non-adherence to the guide (potential DRP) according to the RRMQG was found in 54 of the 378 prescriptions (14.3%). Of these, eight medications were paused at the time of data collection. The potential DRPs were observed within all ATC categorizations except ATC codes L and R. These were rarely prescribed and accounted for only four medications (approximately 1%). In total, 211 (55.8%) of the identified RRMs adhered to the recommendations of RRMQG, with 3 medications paused at the time of data collection; thus, no PCNE-DRP-classification could be applied. The recommendation according to the RRMQG was to monitor the medication (Table 3).

For the remaining 113 (28.9%) RRMs, the clinical pharmacist reported adherence to the RRMQG with no recommendation necessary. However, it was not possible to confirm this assessment by the three assessment pharmacists due to missing information on dose, dosing interval, indication, or, in the case of Mirabegron, information about CYP3A4 inhibitors in the medication list.

### 3.5. Type of PCNE-DRP Identified

The 54 potential DRPs from the RRMQG were categorized according to the PCNE Classification DRP primary domains and potential causes: drug selection (contraindicated, no indication, inappropriate choice of drug) and dose selection (dosage regimen too frequent, reduce dose, increase dose) (Table 4). The most common type of potential causes was “Contraindicated”, accounting for 33 of the 54 DRPs (61.1%), while “Inappropriate choice of drug” accounted for 10 of the 54 DRPs (18.5%). Of the 33 contraindicated medications, 6 were paused following the medication review, as well as 1 medication categorized as “Inappropriate choice of drug”, and 1 medication categorized as “Reduce dose”.

### 3.6. Hospital Readmission and Mortality Among Patients Treated with RRMs

The clinical evaluation showed a significantly higher readmission rate among males, *p* = 0.012 (χ^2^-test) (Table 5). None of the remaining variables showed significant differences between no readmission, readmission or mortality. Patients who died or were readmitted within 30 days of discharge had a tendency toward lower eGFR and more potential renal risk DRPs compared to patients that were not readmitted; however, the differences were non-significant (Table 5).

Although only patients with a plasma creatinine measurement within 30 days prior to hospital admission were included, aiming to avoid inclusion of patients with AKI, 19.8% (*n* = 36) of the cohort presented with AKI according to the calculations described above. Generally, AKI (*n* = 36) and CKD (*n* = 146) patients held similar patient characteristics (age, BMI, number of medications and comorbidities), creatinine levels, and eGFR. However, the percentages of RRMs (17.6% versus 5.1%), DRPs (44.4% versus 26.0%), and mortality within 30 days (27.8% versus 15.8%) were higher for AKI patients compared to CKD patients. Conversely, the number of readmissions was slightly higher in CKD patients compared to AKI patients (30.1% versus 22.2%), whereas no difference was seen for “no readmission”.

### 3.7. Clinical Pharmacist Evaluation of the Useability and Quality of the RRMQG

All 28 clinical pharmacists that participated in data collection responded to the evaluation survey (Table 6). The group of clinical pharmacists was heterogeneous in terms of experience with clinical tasks in a hospital pharmacy with an even distribution between experience categories (Table 6).

The majority (57%) of the clinical pharmacists found the RRMQG useful, and the proportion was similar across all categories of experience. One clinical pharmacist did not find the guide useful (Table 6). Some (29%) of the clinical pharmacists reported that the quality of their medication reviews had improved after using the RRMQG, with a higher proportion among clinical pharmacists with little experience compared to the more experienced pharmacists (Table 6).

For the remaining pharmacists (71%), the lack of improvement in recommendations from the RRMQG was due to a limitation in the number of RRMs, RRM-centered pharmacy practice already being established, it being too theory-based, etc. (Table 6).

More than half of the pharmacists (61%) reported that the RRMQG was also relevant to other healthcare professionals besides pharmacists, such as (junior) doctors, nurses and healthcare professionals involved in the medication process. The majority (75%) of the clinical pharmacists involved in data collection reported, regardless of experience level, that they considered permanent implementation of the RRMQG to be manageable (Table 6).

## 4. Discussion

This study showed that the RRMQG was useful in practice. The included 182 patients had a total of 378 RRMs, and 14% of the these were associated with a potential DRP. These could be avoided by dose adjustment based on the RRMQG as part of medication reviews. The majority of the clinical pharmacists reported that the RRMQG was useful for implementation in practice.

### 4.1. Relevance of the RRMQG in Practice

The RRMQG seemed relevant for application among the 182 included patients who were treated with 35 of the 50 RRMs on the list. This result was expected due to the inclusion criteria of patients in the current study (impaired renal function, currently prescribed at least one RRM, etc.) and the RRMQG containing the most frequently used RRM based on the following three criteria: 1: recommendation for medication adjustment in kidney disease as stated in the source; AND 2: ≥30% of the medication or its active metabolites are renally excreted; OR 3: contraindication in patients with impaired renal function [12].

A recent systematic review including 27 studies revealed that the prevalence of inappropriate prescribing among patients with impaired renal function varied between populations: hospital settings (12.6–96%), outpatient settings (0.3–66%) and non-hospital settings (3.9–60%) [19]. The systematic review emphasized the high prevalence of inappropriate prescribing among hospitalized patients with impaired renal function and the clinical importance of patient-specific interventions, which was evident in the current study [19]. Indeed, 14.3% of the prescribed RRMs were categorized as a DRP requiring changes to the prescribed medication regimen. This finding is consistent with results reported in the systematic review; however, the included studies varied considerably with regard to methodology and hence levels of recommendations reported [19].

Feedback about the practical relevance from the 28 participating clinical pharmacists revealed that the majority found the RRMQG manageable for implementation in practice, especially due to providing an overview and focus on RRM as a part of medication reviews. In that way, the most relevant information can be accessed through one source, which makes the daily routines easier, since many different sources and clinical guidelines are available for the optimal treatment of RRMs. The RRMQG was found to be moderately useful, and some clinical pharmacists reported that the RRMQG improved the quality of their medication reviews; however, this was most evident among those with little clinical experience. This may be due to more experienced clinical pharmacists having established a routine for assessing RRMs as a part of conducting medication reviews. In those cases, the RRMQG will provide a better overview but not necessarily improve the quality of the medication reviews, although one pharmacist revealed that the RRMQG may impact the time spent on conducting a review positively.

In addition, qualitative statements revealed that to some, the RRMQG can be used as a basis for each ward pharmacist to adjust RRMs accordingly at their department to meet RRMs used in that clinical setting. Further, it was also suggested that the RRMQG was found to be relevant as an introduction to RRMs in clinical practice for new employees and/or students in general (pharmacists, medical, etc.). Further, other hospital personnel (pharmaconomist or dispensing assistant: In Denmark, a pharmaconomist education is a three-year program consisting in practical training periods and theoretical courses. They hold a higher level of qualification than a general European pharmacy technician does, more comparable to that of a bachelor’s degree. In Denmark, pharmaconomists make up the majority of the staff in both community and hospital pharmacies [20]) were also mentioned as benefiting from the guide for screening purposes during the preparation of medication for admitted patients. Thus, the RRMQG may be more suitable to increase awareness of RRMs for patients with impaired renal function, but for the majority of clinical pharmacists, they did not find a benefit for themselves by using the guide. There may also be an ethical dilemma involved where it can be difficult to self-evaluate if you do not have the knowledge, skills or focus on impaired renal function before the project initiation. However, the pharmacists already seemed to have an increased focus on RRMs and impaired renal function; thus, the list may be redundant in their established work procedures, but the implementation of the RRMQG was found to be manageable and valuable for the pharmacists that do not have an increased focus on RRMs.

### 4.2. The RRMQG Compared to Other Recommendations

The RRMQG comprises the most frequently used RRMs in primary and secondary care in Denmark. This makes the RRMQG relevant and easily applicable; however, it turns out that an important group of medications was not included in the list due to the methodology used developing the list, namely analgesics, such as morphine, oxycodone and tramadol [12]. For example, when specific wards use opioids routinely, such as orthopedic wards for pain treatment after hip replacement surgery, the RRMQG is less useful in that matter. However, these opioids are listed on another Danish list of RRMs developed for use in general practice [21]; hence, it is advisable to expand the RRMQG with these medications. Another strategy could be to adjust the RRMQG to individual wards based on their medication use and by that means improve the usefulness of the RRMQG.

Opioids are also considered as one of the Beers criteria for potentially inappropriate medication use in older adults; however, these criteria are difficult to use in the Danish and European setting due to many medications only being available on the American market [22]. In addition, the Beers criteria provide information for 19 medications that require dose adjustments based on eGFR, but they often lack recommendations for clinicians regarding the individual medications on the list.

Similarly, the European Screening Tool of Older People’s Prescriptions (STOPP) and Screening Tool to Alert to Right Treatment (START) Criteria, version 3 from 2023, offer dosing recommendations for 16 medications requiring eGFR-based adjustments [23]. Opioids are mentioned as a part of the STOPP criteria; however, this is in relation to risk of falls, and no recommendation for clinicians was mentioned here either. Indeed, the benefits of the Beers criteria and the STOPP/START criteria have been extensively reported in the literature [24,25,26,27,28], but they lack specific focus on RRMs prescribed to patients suffering impaired renal function. The RRMQG is based solemnly on the 50 most used RRMs prescribed in a Danish context in both primary and secondary care [12]. The purpose is to draw attention to patients with impaired kidney function, as this is a particularly vulnerable patient group, which this pilot study also demonstrates. Further, the list should allow a rapid lookup of specific medications with respect to recommendations based on kidney function. However, the RRMQG is not intended to be used in isolation but rather to use the list as support to other tools that do not share the same specific focus, e.g., the Beers criteria and the STOPP/START criteria. As stated by the systematic review by Hamzaei et al., explicit criteria like the RRMQG, STOPP/START criteria and Beers criteria are not sufficiently effective when used on their own as computerized alerts [19]. They should be included in intervention models based on human involvement focusing on patient-specific interventions for patients with CKD.

### 4.3. Types of DRPs

Of the RRMs identified in this study, 54 (14.3%) were categorized as a DRP, requiring changes to the prescribed medication regimen. The most common type of DRPs was “Contraindicated” (61%), followed by “Inappropriate choice of drug” (19%). These DRPs are considered quite severe, which emphasizes the need for attention among this high-risk group of patients. The types of DRPs most frequently found are similar to the types reported in the literature [19,29,30,31].

The medications causing the DRPs were most frequently a diuretic; bendroflumethiazide/K^+^, followed by a calcium antagonist; lercanidipine; anticoagulants; and cholecalciferol. Again, that is consistent with the literature identifying cardiovascular-acting agents and antithrombotic medications among the most frequently used medications by patients suffering renal impairment [19,29,31]. This indicates that medications frequently used by this population are also the medications often causing DRPs. A further overlap in drug classes from potential DRPs identified using the RRMQG and inappropriate prescribing in other studies were related to anticoagulants/antithrombotics; cardiovascular agents; analgesics and antidiabetics [29,30,31,32]. Thus, the clinical use of the RRMQG in practice to prevent DRPs in patients with impaired kidney function is supported by other studies, and they highlight the challenging nature of optimally prescribing and dosing RRMs for those patients [29,30,31,32].

It was advised that the majority of the RRMs (56%) be monitored, which is a relevant recommendation during admission and in general practice. Furosemide led in 90 cases (43%) to the categorization of monitoring, while no other categories were applicable to furosemide. All patients in this study present with impaired kidney function, and some function could be reestablished by treatment with furosemide. This, in combination with the fact that overdosing has not been reported, questions the relevance of including furosemide in the RRMQG. Apixaban was also frequently categorized as “Monitor”; however, other PCNE-DRP classifications were applicable to this medication, and severe bleeding has been reported as a consequence of overdosing. Hence, Apixaban should therefore remain in the RRMQG. However, in the literature, there are a few medicines that are inappropriate to prescribe to patients with impaired kidney failure, which are not as clearly visible in our study: sulfonylureas, memantine and nitrofurantoin [29,31]. These could potentially be added or highlighted more clearly in future versions of the RRMQG, as they recur in CKD as problematic drugs in studies. Thus, combined with the input from missing RRMs from the clinical pharmacists, an update of the RRMQG would be in accordance with relevant literature in clinical practice.

Some RRMs were paused at the time of data collection, which indicates that the treating physician had already identified a potential DRP and acted upon it. However, they were still classified as potential DRPs as they are at risk of being activated again (for example, during transfer to other wards or at discharge) as they are not discontinued from the patients’ medication list. Thus, paused RRMs were important to classify and highlight, as they had the potential of becoming a DRP. Further, the majority of RRMs were not paused, which reveals the importance of clinical pharmacists conducting medication reviews with a focus on RRMs.

### 4.4. Clinical Evaluation

According to the literature, RRMs are associated with higher risk of hospitalization, higher bleeding rates and high risk of all-cause mortality [16,19,29,30]. This is consistent with the types of DRPs identified in the current study. Indeed, despite this study being an exploratory pilot study, a clinical evaluation was performed for variables indicating large differences between no readmission, readmission to hospital within 30 days and mortality. As the only variable, a significantly higher readmission rate among males treated with RRMs was detected, but it is difficult to assess whether this study has sufficient statistical power to detect an effect, as no prior power calculation was performed. However, this emphasizes that extra attention should be given to males treated with RRMs with regard to optimization of their medication treatment, which is also reported in another study concerning clinical pharmacist intervention in hospital [15].

Patients who died or were readmitted within 30 days of discharge were associated with a lower eGFR and a trend toward higher DRPs compared to patients with no readmission. This draws attention towards the need to focus on the medical treatment for patients with poor eGFR in an attempt to prevent or postpone adverse events. However, potential causal inferences are made with extreme caution, as we acknowledge that interpretation of causal inferences would require a controlled or comparative study design.

Although patients with AKI were initially intended to be excluded, complete exclusion was not feasible in practice. In most cases, plasma creatinine measured immediately before discharge decreased by more than 50% compared with levels prior to or during hospitalization, suggesting recovery from an acute episode. In other cases, plasma creatinine increased by at least 50% during admission, consistent with the development of AKI. Consequently, nearly 20% of patients with AKI remained in the dataset and were included in the evaluation of the applicability of the RRMQG. This inclusion of AKI patients has important implications for the interpretation of our findings. First, eGFR in AKI patients is typically more unstable and prone to rapid change than in patients with stable CKD, which may increase the risk of DRPs associated with RRMs [33]. Although the overall effect of including AKI patients is difficult to quantify, our results indicate that the number of RRMs and DRPs was higher in AKI patients compared to those with CKD. The same pattern was observed for mortality, in line with previous studies [34,35], reporting that AKI is associated with increased risks of cardiovascular mortality, CKD, CKD progression and end-stage renal disease [34]. However, in the present study these differences did not reach statistical significance.

Second, the inclusion of AKI patients increases both the clinical and prognostic heterogeneity of the study population. This may have reduced the precision and interpretability of the estimated effects, as patients with stable CKD and those experiencing AKI can have markedly different risk profiles, disease trajectories and responses to renal risk-modifying interventions. In addition, AKI and its associated clinical factors (e.g., higher comorbidity burden, acute illness, medication changes) may act as confounders, being related both to the likelihood and nature of interventions and to outcomes such as kidney disease progression, hospitalization and mortality. Therefore, residual confounding cannot be excluded, and the generalizability of our findings to a population with purely stable CKD is limited and should be interpreted with caution.

However, we have deliberately included patients with AKI in these analyses, even though this increases clinical and prognostic heterogeneity and limits the generalizability to a purely stable CKD population, because it reflects real-world clinical practice and allows for a more pragmatic assessment of the applicability of the RRMQG.

### 4.5. Strengths and Limitations

A major strength of this study was participation of clinical pharmacists from all Danish regions, which supports the results that the RRMQG was manageable for implementation in clinical practice. The first limitation of this study was reports with incomplete data. This may rely on the heterogeneity in experience among the clinical pharmacists who collected the data both with regard to conducting research projects and with clinical practice, including performance of medication reviews. The incomplete data on the type of RRMs and the subsequent confirmation process by the assessment pharmacists on adherence to RRMQG were therefore unattainable. Thus, this could potentially be a source of underreporting of DRPs. The second limitation in this study was the lack of focus on the identification of potential DRPs to physicians, particularly in regard to acceptance or rejection of potential pharmacist recommendations to RRMs. This limits the assessment of the RRMQG’s impact on patient care. Another limitation was difficulties distinguishing between AKI and CKD at the time of patient inclusion, as already mentioned. Lastly, the lack of validation to confirm adherence to RRMQG (categorized as “no recommendation” from clinical pharmacists) due to missing information (dose, dosing interval, indication) is a limitation in this study. However, it is not considered critical in the categorization of potential DRPs, as the clinical pharmacists were instructed to use the RRMGQ and to report potential recommendations for RRMs (strength, dose, indication, etc.). However, no recommendations and further details were reported for 113 out of 378 RRMs, which supports the assumption of adherence to the RRMQG in these situations. Nonetheless, this is a data completeness issue and thus a limitation in this exploratory study.

The majority of participating clinical pharmacists were strongly experienced in conducting medication reviews; however, we did not assess the impact of recommendations regarding RRM made by clinical pharmacists since the current study was designed as an exploratory study. Further, no power calculation was conducted, as the focus of this study was usefulness in practice. However, we chose to perform a few statistical analyses, because differences in the results seemed quite large. Yet, the findings from the analysis are interpreted with caution, as we are fully aware of the methodological limitation potential that insufficient power presents in this study. Hence, this pilot study calls for a larger clinical study focusing on outcome assessment and causal inference, where the findings of the current study can be tested statistically in a controlled or comparative study design. For further development, the RRMQG should be: (1) modified based on the feedback from clinical pharmacists; (2) critically reviewed in terms of the relevance of recommendations in clinical practice; (3) have its recommendations combined with the Beers criteria, STOPP/START criteria, the Danish Kidney Medication List for General Practice and the relevant literature concerning the prescribing of RRMs to patients with impaired kidney function.

In future research, the usefulness of RRMQG should be explored as a part of the introduction to clinical practice to students and/or new employees involved in the medication process across professions. Furthermore, pharmaconomists or dispensing assistants should also be introduced to the RRMQG for screening purposes during the preparation of medication phase for patients with impaired renal function. All of the initiatives will support any clinical practice that focusses on renal risk medications for patients with renal impairment both nationally and internationally.

## 5. Conclusions

This exploratory multicenter study suggests that the RRMQG is a manageable and practically applicable tool to support medication reviews for patients with impaired kidney function in Danish hospital pharmacies. The RRMQG appears particularly valuable in settings and among professionals who do not already have a strong focus on RRMs, and it may also aid pharmaconomists/dispensing assistants during the medication process. By concentrating on the most frequently used RRMs and providing rapid, eGFR-based recommendations, the RRMQG complements existing tools, which often lack a specific renal focus.

This study identified a substantial proportion of clinically relevant DRPs, especially contraindications and inappropriate drug choices, often involving cardiovascular agents, antithrombotics and diuretics, underlining the vulnerability of this patient group and the importance of systematic RRM reviews. Additionally, the findings highlight the need to update the RRMQG by including the addition of frequently used opioids and reconsidering the relevance of some current entries such as furosemide.

Although methodological limitations and the inclusion of both CKD and AKI patients preclude causal conclusions, the trends observed support further controlled studies to evaluate the clinical impact of the RRMQG, particularly when integrated into multidisciplinary, patient-centered intervention models and used in the training of new staff and students.

## Figures and Tables

**Table 1:** Baseline characteristics for all included patients.

Demographics	Value
Female sex	95 (52.2%)
Age median, IQR	82.0 [78.0; 88.8]
BMI (*n* = 163) median, IQR	25.1 [22.5; 28.6]
eGFR at medication review (mL/min/1.73 m^2^)	34 [23; 43]
Number of medications	14 [12; 18]
Number of renal risk medications	2.0 [2.0; 3.0]
Comorbidities median, IQR	2 [1; 3]
Cardiac	167 (91.8%)
Diabetes	56 (30.8%)
Pulmonary	47 (25.8%)
Musculoskeletal disorders	29 (15.9%)
Psychiatric	21 (11.5%)
Cancers (current treatment)	20 (11.0%)
Dementia	18 (9.9%)
Region of Southern Denmark	80 (44.0%)
Central Denmark Region	13 (7.1%)
Northern Denmark Region	20 (11.0%)
Region Zealand	34 (18.7%)
Capital Region of Denmark	35 (19.2%)

Values are presented as counts (%) unless otherwise specified. IQR: [interquartile range].

**Table 2:** Frequency of renal risk medications from the RRMQG.

ATC ^1^ 1st Level		ATC 2nd Level	
	Number of medications		Number of medications
A	39 (10.3%)	A10	26
		A11	13
B	64 (16.9%)	B01	64
C	185 (48.9%)	C01	8
		C03	119
		C08	14
		C09	33
		C10	11
G	2 (0.5%)	G04	2
J	22 (5.8%))	J01	22
L	3 (0.7%)	L02	1
		L04	2
M	7 (1.9%)	M01	3
		M05	4
N	55 (14.6%)	N02	38
		N03	2
		N05	6
		N06	9
R	1 (0.3%)	R06	1

RRMs from the RRMQG presented as ATC at 1st and 2nd levels (number of medications = 378). The data include all prescribed medications from the list, and paused medication was not omitted (*n* = 157). RRM: renal risk medication. RRMQG: renal risk medication quick guide. ^1^ ATC main groups: A (alimentary tract and metabolism), B (blood and blood-forming organs), C (cardiovascular system), G (genito-urinary system and sex hormones), J (anti-infectives for systemic use), L (antineoplastic and immunomodulating agents), M (musculoskeletal system), N (nervous system) and R (respiratory system).

**Table 3:** Frequency and categorization of renal risk medications from the RRMQG.

Patients’ Medications from the RRMQG			Number of Medications (Paused)		
ATC 2nd Level	Medication	Number of Medications	PCNE-DRP	No PCNE-DRP; Monitor	No Recommendation
A10	Empagliflozin	11	4	1	6
	Metformin	15	3	2	10 (1)
A11	Cholecalciferol	13	6	7	-
B01	Apixaban	33	3 (1)	19 (1)	11
	Dabigatran etexilat	2	-	-	2
	Dalteparin	9	2	3	4
	Rivaroxaban	18	2 (2)	5 (1)	11
	Tinzaparin	1	-	-	1
C01	Digoxin	8	-	-	8
C03	Bendroflumethiazide/K^+^	15	6 (1)	9	-
	Furosemide	90	-	90 (1)	-
	Spironolactone	16	4 (2)	4	8 (1)
C08	Lercanidipin	14	6 (2)	8	-
C09	Enalapril	17	-	16	1
	Lisinopril	1	-	-	1
	Ramipril	15	1	14	
C10	Rosuvastatin	10	-	2	8 (1)
G04	Mirabegron	2	-	-	2
J01	Piperacillin/beta-lactamase inhibitor	17	3	5	9
	Pivmecillinam	5	4	1	-
L02	Anastrozol	1	-	1	-
L04	Azathioprin	1	-	1	-
	Mycophenolsyre	1	-	1	-
M01	Ibuprofen	3	2	1	-
M05	Alendronsyre	4	4	-	-
N02	Gabapentin	17	1	2	14
	Pregabalin	8	-	1	7
	Tramadol	13	-	10	3 (1)
N03	Levetiracetam	2	-	-	2
N05	Melatonin	4	1	1	2
	Olanzapin	2	-	1	1
N06	Citalopram	5	-	4	1
	Duloxetin	2	2	-	-
	Venlafaxin	2	-	2	-
R06	Cetirizin	1	-	-	1
Total		378	54 (8)	211 (3)	113 (3)

RRMs and potential identified DRPs divided into three categories: PCNE-DRP (non-adherence to RRMQG), monitoring (adherence to RRMQG, but monitoring activity recommended) and “no recommendation” (not possible to categorize from medication review). RRM: renal risk medication. RRMQG: renal risk medication quick guide. PCNE: Pharmaceutical Care Network Europe. DRP: drug-related problem.

**Table 4:** Potential DRP causes according to PCNE Classification for Drug-Related Problems V9.1.

PCNE-DRP Causes	Drug Selection			Dose Selection			
Medication	Contraindicated	No Indication	Inappropriate Choice of Drug	Dosage Regimen Too Frequent	Reduce Dose	Increase Dose	Number of Medications (Paused)
Empagliflozin	3				1		4
Metformin	3						3
Cholecalciferol	6						6
Apixaban	2 (1)					1	3 (1)
Dalteparin					2		2
Rivaroxaban	2 (2)						2 (2)
Bendroflumethiazide/K^+^			6 (1)				6 (1)
Spironolactone	2 (1)				2 (1)		4 (2)
Lercanidipine	6 (2)						6 (2)
Ramipril		1					1
Piperacillin/beta-lactamase inhibitor				3			3
Pivmecillinam			4				4
Ibuprofen	2						2
Alendronic acid	4						4
Gabapentin					1		1
Melatonin	1						1
Duloxetine	2						2
TOTAL	33 (6)	1	10 (1)	3	6 (1)	1	54 (8)

PCNE: Pharmaceutical Care Network Europe. DRP: drug-related problem.

**Table 5:** Hospital readmission and mortality among patients treated with renal risk medications.

	No Readmission (*n* = 96; 53.0%)	Hospital Readmission (*n* = 52; 28.7%)	Mortality (*n* = 33; 18.2%)
Female sex; *n* (%) Male sex; *n* (%)	57 (61%) 39 (45%)	18 (19%) 34 (39%)	19 (20%) 14 (16%)
Age	82.0 [78.0; 88.0]	80.0 [76.0; 87.5]	82.0 [78.0; 89.0]
BMI (*n* = 163)	25.7 [23.3; 29.4]	24.8 [21.6; 29.3]	24.8 [21.2; 27.6]
eGFR at medication review (mL/min/1.73 m^2^)	37 [25.8; 44.0]	31.0 [21.8; 43.3]	28.0 [15.0; 42.0]
Number of medications	14 [12; 18]	16 [12; 19]	13 [10; 17]
Number of renal risk medications	2.0 [2; 3]	2.0 [2; 4]	2.0 [2; 3]
Comorbidities	2.0 [1; 3]	2.0 [1; 3]	2.0 [1; 2]
DRPs per patient	0.2 per patient	0.4 per patient	0.4 per patient

Values are presented as median [IQR] for continuous variables and counts (%) for categorical variables (*n* = 181). DRP: drug-related problem.

**Table 6:** Clinical pharmacists’ evaluation of the useability of RRMQG.

Years of Experience with Clinical Tasks at Hospital Pharmacy, Such as Medication Review	0–2 y	3–5 y	6–9 y	>10 y	Sum; n (%)
Number of clinical pharmacists	7	7	9	5	28
The usefulness of the RRMQG					
Very useful/Useful; n	5	4	4	3	16 (57%)
Little or rarely useful; n	1	3	5	2	11 (39%)
Not useful at all; n	1	-	-	-	1 (4%)
Please elaborate (optional): “The list has enhanced the focus on risk medication and impaired kidney function” [6–9 years of experience] “The list creates a better overview of medications, which may be problematic in the treatment of patients with impaired kidney function. It provides a quick overview of potential recommendations in terms of indication, contraindications, dose adjustments etc.” [0–2 years of experience] “Gathering information on medications, which may have a consequence with impaired renal function, has improved attention to this challenge when conducting medication reviews” [6–9 years of experience] “The quality of my medication reviews is probably unchanged, but the advantage of using the list is that I now only need to use one source of information” [3–5 years of experience] “I have become aware of renal risk medications, which I would normally not react upon” [0–2 years of experience]					
Has the RRMQG improved the quality of your recommendations from the medication review?					
Yes; n	4	1	3	0	8 (29%)
No; n	3	6	6	5	20 (71%)
Please elaborate (optional): “The list needs to be more practice-oriented [0–2 years of experience] “The list did not contain any new knowledge” [3–5 years of experience] “We’re allways aware of dosing compared to kidney function” [3–5 years of experience] “I don’t think the quality of my review has improved with the list, but I may conduct reviews quicker because I don’t need to look-up all RRMs [in terms of dosing] elsewhere” [3–5 years of experience] “I don’t think the list could be better, but easier options for look-ups [concerning RRMs] exists” [6–9 years of experience] “I’m aware of majority of the recommendations, but a less experienced pharmacist would probably benefit from the List” [>10 years of experience] “To me, the list does not include the most widely used RRMs” [>10 years of experience]					
Do you find the RRMQG relevant to other healthcare professionals?					
Yes; n	4	5	5	3	17 (61%)
No; n	3	2	4	2	11 (39%)
“The list could be a tool to everyone, who is involved with medicine” [0–2 years of experience] “Certainly it would be useful to doctors and nurses” [3–5 years of experience] “Perhaps it [RRMQG] would be more suitable to other healthcare professionals than pharmacists” [3–5 years of experience] “We [at our hospital] use other look-up guides [than the RRMGQ]” [6–9 years of experience] “I work closely with junior doctors—the list would be useful to them” [>10 years of experience]					
Do you consider a permanent implementation of the RRMQG in your medication review as manageable in hospital pharmacy practice?					
To a high or some extent; n	5	5	7	4	21 (75%)
To a minor extent; n	2	1	2	-	5 (18%)
Almost not; n	-	1	-	1	2 (7%)

RRMQG: renal risk medication quick guide.

## Data Availability

The datasets presented in this article are not readily available due to restrictions in study approvals concerning data-sharing limitations. Reasonable requests to access the datasets should be directed to the corresponding author.

## Supplementary Materials

The following supporting information can be downloaded at: https://www.mdpi.com/article/10.3390/healthcare14091245/s1, Supplementary File S1: Survey to the clinical pharmacists.
