# Supplementary material for: Renal Risk Medication Quick Guide to Aid Pharmacist-Led Medication Review in Frail Hospitalized Geriatric Patients: A Multicenter Exploratory Study
Source: Healthcare (Basel). 2026 May 5;14(9):1245. doi: 10.3390/healthcare14091245 (PMC13163804; doi:10.3390/healthcare14091245)
Supplement: Supplementary file 1 [file healthcare-14-01245-s001.zip › healthcare-4185205-supplementary.pdf]

Supplementary File S1_ Survey to the clinical pharmacists: evaluation of the feasibility of RRMQG

Q1: For how long have you had clinical tasks at hospital pharmacy, e.g. conducting medication review				
0-2 y	3-5 y	6-9 y	>10 y	
Q 2: How useful do you find the RRMQG concerning conducting medication reviews?				
Very useful	Useful	Little useful	Rarely useful	Not useful at all
Please elaborate (optional)				
Q 3: Do you find the number of medications in RRMQG appropriate?				
Yes	No			
Q 4: Compared to you regular medication review, has the RRMQG improved the quality of your recommendations in the medication review?				
Yes	No			
Please elaborate (optional)				
Q 5: Do you find the RRMGQ relevant to other healthcare professionals?				
Yes	No			
Please elaborate (optional)				
Q 6: Do you consider a permanent implementation of the RRMQG in your medication review as feasible in hospital pharmacy practice?				
To a very high extent	To a high extent	To some extent	Barely not	Not at all
Q 7: Have you learned something academically/professionally relevant during this study?				
To a very high extent	To a high extent	To some extent	Barely not	Not at all
